# Heavy metal concentrations and clinical symptoms in patients diagnosed with schizophrenia related to cigarette smoking

**DOI:** 10.1038/s41598-024-64333-9

**Published:** 2024-07-02

**Authors:** Amir Ghaderi, Amir Hossein Khoshakhlagh, Agnieszka Gruszecka-Kosowska, Fatemeh Askari-Lemjiri, Fatemeh Alemi, Nader Molavi, Pooya Hazegh, Bahareh Farokhi, Somayeh Ghadami Dehkohneh, Fatemeh Sadat Ghoreishi

**Affiliations:** 1 Department of Addiction Studies, School of Medical and Clinical Research Development Unit-Matini/Kargarnejad Hospital, Kashan University of Medical Sciences, Kashan, Iran; 2 Department of Occupational Health Engineering, School of Health, Kashan University of Medical Sciences, Kashan, Iran; 3 Faculty of Geology, Geophysics, and Environmental Protection, Department of Environmental Protection, AGH University of Krakow, Al. Mickiewicza 30, 30-059 Krakow, Poland; 4 Student Research Committee, Kashan University of Medical Sciences, Kashan, Iran; 5 Department of Toxicology and Pharmacology, School of Pharmacy, Mazandaran University of Medical Sciences, Sari, Iran; 6 Department of Psychiatry, Kashan University of Medical Sciences, Kashan, Iran; 7 Department of Clinical Psychology, Allameh Tabataba’i University, Tehran, Iran; 8 Department of Pharmacy, Acharya BM Ready College of Pharmacy, Rajive Gandhi University of Health Sciences, Banglore, Karnataka India

**Keywords:** Schizophrenia, Blood, Urine, Arsenic, Cadmium, Lead, Thallium, Biological techniques, Diseases

## Abstract

In our study, blood concentrations of lead (Pb), arsenic (As), and cadmium (Cd) and urine concentrations of thallium (Tl) were measured together with related symptoms of heavy metal poisoning in cigarette smoking volunteers diagnosed with schizophrenia, in cigarette smokers not diagnosed with schizophrenia, and in the control group of non-smokers and not diagnosed with schizophrenia volunteers. Our study was performed on 171 volunteers divided into the following subgroups: patients diagnosed with schizophrenia with at least 1 year of continuous cigarette smoking experience (56 participants), cigarette smokers not diagnosed with schizophrenia with at least one year of continuous smoking experience (58), and control group (not diagnosed with schizophrenia and non-smoking volunteers) (57). Smoking durations of cigarette smokers diagnosed with schizophrenia and cigarette smokers not diagnosed with schizophrenia are not similar (p = 0.431). Blood Pb, As, and Cd concentrations and urine Tl concentrations were the highest in the subgroup of cigarette smokers not diagnosed with schizophrenia, followed by the subgroup of cigarette smokers diagnosed with schizophrenia, and the control group. Only blood Pb concentrations were significantly higher (probability value p < 0.05) in the group of cigarette smokers not diagnosed with schizophrenia (5.16 μg/dL), comparing to the group of cigarette smokers diagnosed with schizophrenia (3.83 μg/dL) and to the control group (3.43 μg/dL). Blood Cd and As concentrations and urine Tl concentrations were significantly higher (p < 0.05) in cigarette smokers not diagnosed with schizophrenia than in the control group. The results revealed a statistically significant positive correlation (p < 0.001) in the cigarette smokers in the schizophrenia diagnosed group between blood Pb, blood As, and urine Tl concentrations and the duration of cigarette smoking.

## Introduction

Today, smoking is considered a worldwide health problem, so in 2021 it was reported to cause one third of all adult deaths^1^. According to the statistics of the World Health Organization (WHO), there are 1.3 billion smokers in the world, of which approximately 8% are from developing countries. The same organization reported the prevalence of smoking in Iran in 2010, 22% in men and 1% in women, and estimated that this rate will reach approximately 19% in men and 10% in women by 2025, and 9% of the Iranian population will be smokers by 2025^2,3^. On the other hand, the WHO estimated that tobacco use (cigarettes and smoke) is currently responsible for the death of 6 million people worldwide and many premature deaths, as well as for one third of all cancers that lead to death, are related to smoking^4,5^. Chronic smoking entails major risks, among which the acute exacerbation of chronic obstructive pulmonary disease, asthma, and the imbalance between blood oxidants and antioxidants^6^ can be mentioned. The range of risks is not only related to the chronic effects of smoking, in fact, acute exposure to cigarette smoke can also lead to many body damages, such as severe tissue damage^7^. These raised issues are proof that no level of smoking is safe and, in both acute and chronic cases, it entails irreparable risks^8^.

Chemical and industrial pollutants endanger human life directly and indirectly. Among these pollutants, heavy metals are of the great concern^9^. Heavy metals have a higher density than other metal elements and their average density is greater than 5 g per cubic centimeter^10^. Today, one of the health and environmental issues of industrial societies is the control of heavy metal pollution, as due to their wide usage sources of their emissions are considered a serious problem for public health^11^. Heavy metal emissions in the environment are caused mainly by human activities and to a much lesser extent by natural events^12^. Among these heavy metals, few affect the human body. Although some of these metals affect human physiology only in high doses, others such as cadmium (Cd), mercury (Hg), lead (Pb), chromium (Cr), silver (Ag), and arsenic (As) have delirious effects on the body even in small amounts and cause acute and chronic poisoning in humans^13^. It has been reported that the tobacco plant easily takes metals from the soil and concentrates them in the leaves. This contamination is different in each country where the tobacco plant is harvested and processed, however, most of the global tobacco leaves produced are concentrated in the People’s Republic of China, the Republic of India, Brazil, the United States of America, and Zimbabwe^14^. About 4700 compounds, many of which are toxic, such as aromatic hydrocarbons, aldehydes, ketones, and heavy metals, are found in tobacco^15^. Smoking is directly and indirectly related to various types of cancer (larynx, esophagus, trachea, lung, kidney, bladder, etc.) and chronic diseases (coronary artery disease, asthma, chronic obstructive pulmonary disease COPD, etc.)^16^. Of about 8000 toxic compounds present in tobacco smoke, the following belong to the group of heavy metals: Cd, Hg, Pb, As, beryllium (Be), nickel (Ni), hexavalent chromium Cr(VI), copper (Cu), barium (Ba), titanium (Ti), and aluminum (Al)^17,18^. Despite the biological plausibility of the link between heavy metal exposure and mental health disorders, epidemiological evidence is scarce^19^.

Psychosis is a serious mental disorder that affects the brain and means an abnormal mental state in which the affected person does not have the ability to recognize reality or fantasy, the most obvious characteristic of this type of mental disorder is disorder in thinking and speech, delirium, and hallucinations^20^. A psychotic disorder usually constitutes more persistent and pervasive psychotic symptoms along with a number of other deficits. One of the most severe psychotic disorders is schizophrenia. Schizophrenia is chronic brain disease that is revealed in disorders of thought processes, perceptions, emotional response, and social interactions^21^. It has long been recognized that there is a strong relationship between smoking and psychotic disorders. Recently, smoking has also been found to be associated with psychotic experiences in the general population^22–25^. The link between smoking and schizophrenia is well established. The amount of smoking in people with psychotic disorders is 2–3 times higher than in people without disorders^24^. In addition, smokers with diagnosed psychotic disorders show high smoking patterns and strong nicotine dependence and are less likely to quit compared to non-smokers^26^. Among various pollutants, the main pollutants that can cause schizophrenia are heavy metals^27^. A relationship between air pollution, heavy metals, and chemicals with psychotic complications, one of which is schizophrenia is also reported in the studies^28^. Air pollution is one of the problems faced worldwide, especially in developed and developing countries^28^. The positive correlations between Pb concentrations in early life and psychotic experiences, such as schizophrenia in adulthood, were reported^29^.

Considering that there is not enough information on the presence of heavy metals in smoking patients diagnosed with schizophrenia and also that the results of other studies revealed that most of Iranian and foreign brands of cigarettes have a high level of contamination with heavy metals in the cigarette filter, especially after smoking, and regarding the widespread use of cigarettes among individuals with schizophrenia and the related toxicity of heavy metal contamination, we investigated the correlation between the concentrations of four heavy metals, namely Pb, Cd, As, and Tl, and related clinical symptoms in the three investigated subgroups, that were cigarette smokers recruited from patients diagnosed with schizophrenia, cigarette smokers not diagnosed with schizophrenia, and the control group constituted of non-smokers and not diagnosed with schizophrenia volunteers. The results of our study can be useful for healthcare organizations in establishing public health guidelines. The results might also provide new knowledge, as best to our knowledge, there is a lack of similar studies in persons with schizophrenia, especially that there is a high probability of the occurrence of the adverse health effects among cigarette smokers.

## Methods

### Participants of the study

Our cross-sectional study was performed from September 2022 to January 2023 in the population aged from 17 to 60 years old. Patients diagnosed with schizophrenia, according to the criteria of Diagnostic and Statistical Manual of Mental Disorders, Fourth Edition (DSM-IV)^30^, having at least one year of continuous daily smoking experience were recruited from those admitted to Karegarnjad Psychiatric Hospital in Kashan, the Islamic Republic of Iran. Cigarette smokers with at least one year of continuous daily smoking experience and not diagnosed with schizophrenia were recruited from laboratories of various departments of Shahid Beheshti Hospital in Kashan, the Islamic Republic of Iran, during routine visits to the doctor, as well as from cafes located in Kashan city. Control group volunteers were made up of individuals not diagnosed with schizophrenia and non-smoking volunteers who were selected from family members of cigarette smokers and diagnosed with schizophrenia patients as having the same living environment, water, and food consumption. In total, in our research, 171 volunteers were examined divided into the three subgroups as follows: (1) patients diagnosed with schizophrenia with at least one year of continuous cigarette smoking experience: 56 volunteers, (2) cigarette smokers not diagnosed with schizophrenia with at least one year of continuous smoking experience: 58 volunteers and (3) control group (not diagnosed with schizophrenia individuals and non-smoking volunteers): 57 volunteers.

To obtain the minimum sample size, using the results of Arinola et al.'s study^31^, the mean ± SD level of lead in healthy and schizophrenic patients using antipsychotic drugs was 44.8 ± 7.25 and 49.5 ± 6.48, respectively, and also considering the 95% confidence interval and 90% test power, the number of 45 people in each group was calculated, with a 15% probability of possible dropout and non-completeness of information, so, the number of 60 people in each group was investigated.

### Inclusion and exclusion criteria

Inclusion criteria were as follows: cigarette smokers both among patients diagnosed with schizophrenia and individuals not diagnosed with schizophrenia who had a history of continuous daily smoking for at least one year before the study, completion of the consent form before starting of the research, and age between 17 and 60 years old. Exclusion criteria were: a history of heavy metal poisoning (self-reported), a working history in occupations related to the presence of heavy metals such as battery making, soldering, painting, crystallization, cement factories, iron smelting factories, power plants, as well as a history of drug use in psychiatric disorders and mental problems among cigarette smokers not diagnosed with schizophrenia and the control group (not diagnosed with schizophrenia and non-smokers). In addition, all participants were primary schizophrenia in this study and we did not include secondary schizophrenia in the current study.

### Data and sample collection

To all recruited volunteers in the three investigated subgroups the research plan was explained, and the informed consent was signed by all participants before starting the study. Research was carried out according to the Declaration of Helsinki and the protocol was approved by the ethics committee of the Kashan University of Medical Sciences (IR.KAUMS.MEDNT.REC.1401.093). After obtaining informed consents, demographic characteristics including data on age, gender, occupation, education, age at first cigarette smoking, type of cigarette brand, reason for smoking, history of smoking in the family, duration of smoking, number of times of smoking daily, the antipsychotic drug used, and the duration of schizophrenia were recorded in the prepared checklist. In addition, the Positive and Negative Syndrome Scale (PANSS) checklist of the clinical symptoms among patients diagnosed with schizophrenia was used. Furthermore, smoke pack year (SPY) measure was used to compare smokers.

In addition to demographic information and the PANSS clinical symptoms checklist, all recruited volunteers completed questionnaires that record the clinical symptoms of heavy metal poisoning (neurological, digestive, and skin symptoms). Project managers asked volunteers in the three subgroups about clinical symptoms and in the questionnaire the presence of symptoms was scored with value 1 and scored with value 0 if no symptoms were reported.

Blood and urine samples were collected from all recruited volunteers in all three investigated subgroups. Considering that the excretion rate of Tl in urine is higher, therefore urine samples were used in order to determine Tl concentrations, while for other heavy metals, namely As, Cd, and Pb, blood serum was used.

### Determining heavy metal concentrations

To measure the concentrations of Tl in urine and As, Cd and Pb in blood serum samples, the graphite furnace atomic absorption spectrometer (GFAAS) (Perkin-Elmer model 3030B; Waltham, Massachusetts, United States of America) was used.

In order to perform analysis on the GFAAS, a suitable detection metal lamp was installed in the device, and the wavelength of the desired slit was adjusted, followed by the adjustment of the graphite furnace in the place of the transition path. For measuring the content of Pb, Cd, and As in blood serum, after clotting with nitric acid by adding Triton X-100, separation of the liquid phases were done from the clot. The heavy metal stabilization to atomization and neutralization of the acidity to prevent damage to the graphite tube modifier was added. In a 1.5 mL microtube the solutions including 200 μL of 5% nitric acid and 200 μL of 3% Triton X-100 were added to 100 μL of standard, blank, and blood samples. These mixtures were vortexed and centrifuged for approximately 2 min. The modifier solution was then added with a concentration of 200 μL was added and centrifuged for 2 min, and then 25 μL of the upper layer was injected into the graphite tube^32^.

For analysis of Tl concentrations, 10 mL of urine was taken in a plastic vessel from a 24-h urine sample of the participant. The Tl measurement in urine was performed using nitric acid and Triton X-100. The solutions including sulfuric acid 1% (0.5 mL), nitric acid (0.5 mL), and Triton X-100 (10 mL) were added to standard, blank, and urine samples. The mixture was vortexed and centrifuged for approximately 2 min. Then, the modifier solution with a concentration of 200 mL was added, and centrifuged for almost 2 min, and 25 mL from the upper layer was injected into the graphite tube. All blank, standard, and control samples were prepared in a similar way. For Tl measurement electrothermal atomic absorption spectrometer (ET-AAS) with the following thermal program was used: for drying the temperature was 130 °C, for the removal of organic compounds the temperature was 300 °C, for the decomposition of inorganic compounds the temperature was 600 °C, and for the atomization the temperature was 1700 °C^33–35^.

The samples were transmitted to the laboratory in less than 2 h and kept at 36 °C until analysis. All laboratory equipment and vessels were rinsed with dilute HNO_3_ (Merck) and washed with ultrapure water (18.2 MΩ cm) prior to usage^36^. All biochemical analyses were evaluated by an expert.

### Statistical analysis

For statistical analysis in our study, Statistical Package for the Social Sciences (SPSS) software version 26 was used. Descriptive statistics included frequency distribution and central and dispersion indices analysis. Inferential statistics included performing of the chi-squared test and analysis of variance, respectively, to compare the relative frequency of qualitative variables and the mean of quantitative variables between groups. The independent Student’s t-test was performed to compare the mean concentration of heavy metals according to the clinical symptoms of heavy metals poisoning. The Pearson's correlation test was used to correlate the heavy metal concentrations with the PANSS scale and the duration of smoking.

### Ethics approval and consent to participate

All participants gave their signed written informed consent letters. The study protocol was approved by the Ethics Committee of Kashan University of Medical Sciences “approval no IR.KAUMS.MEDNT.REC.1401.093". All procedures performed in studies involving human participants were in accordance with the ethical standards of the institutional and national research committee and with the 1964 Helsinki declaration and its later amendments.

## Results

The characteristics of three subgroups of volunteers investigated in our studies are presented in Table 1. Regarding the gender, in all investigated subgroups, most volunteers were men: among cigarette smokers diagnosed with schizophrenia 76.8%, among cigarette smokers not diagnosed with schizophrenia 87.9%, and in the control group (not diagnosed with schizophrenia and non-smokers) 80.7%. The mean age was similar in the investigated subgroups and was around 38.6 years old among cigarette smokers diagnosed with schizophrenia, 35.9 years old among cigarette smokers not diagnosed with schizophrenia, and 36.2 years old in the control group. Most of the cigarette smokers diagnosed with schizophrenia were singles (50%), while in the cigarette smokers not diagnosed with schizophrenia and in the control group, most of the investigated volunteers were married. In cigarette smokers diagnosed with schizophrenia subgroup middle level of education dominated (37.5%). In the cigarette smokers not diagnosed with schizophrenia, the middle and university education level were the most abundant, both counting 32.8% of the respondents. In the control group, the university level of education dominated with 43.9% of respondents. Regarding occupational status in the cigarette smokers diagnosed with schizophrenia, the unemployed subgroup dominated (75% of respondents). In the other subgroups, most of the respondents chose the “other” answer regarding the occupation state: 44.9% of the responses in the cigarette smokers not diagnosed with schizophrenia subgroup and 43.9% of the responses in the control group. Interestingly, in both investigated groups of cigarette smokers, 60% of the respondents declared a history of the smoking in their family (60.7% in the patients diagnosed with schizophrenia subgroup and 60.3% in those not diagnosed with schizophrenia smokers), while in the control group the history of smoking in the family declared only 42.1% of the respondents. The mean age of smoking initiation, the number of cigarettes used daily, and the duration of smoking in years were similar in both investigated groups of smokers. In the subgroup of patients diagnosed with schizophrenia, the mean smoking initiation age was equal to 22.5 years old, the number of cigarettes used daily was declared to be 1 package (20 cigarettes) by 66.1% of respondents, and the mean smoking duration was equal to 12.5 years. In the cigarette smokers not diagnosed with schizophrenia subgroup, the mean smoking initiation age was equal to 20.2 years old, the number of cigarettes used daily was declared to be 1 package (20 cigarettes) by 62.1% of the respondents, and the mean duration of smoking was equal to 13.6 years. In the cigarette smokers diagnosed with schizophrenia subgroup, the smoking exposure is 24.96 pack-years, while in the cigarette smokers not diagnosed with schizophrenia subgroup the smoking exposure is 23.8875 pack-years. The findings of Table 1 showed that the variables of marital status, education level, occupation, and age of smoking initiation were statistically significant and differed between the studied groups (p < 0.05).

**Table 1:** Descriptive characteristics of volunteers in investigated subgroups in the study; data reported as frequency (percentage) or mean ± standard deviation (minimum–maximum); – not applicable; package of cigarettes: 20 pieces of cigarettes.

Variable	Cigarette smokers diagnosed with schizophrenia	Cigarette smokers not diagnosed with schizophrenia	Control group (not diagnosed with schizophrenia and non-smokers)	p-value
Gender				
Male	43 (76.8%)	51 (87.9)	46 (80.7)	0.292*
Female	13 (23.2)	7 (12.1)	11 (19.3)	
Age (years old)	6.33 ± 38.64 (27–55)	10.37 ± 35.93 (23–62)	10.12 ± 36.21 (22–60)	0.137**
Marital status				
Single	28 (50)	22 (37.9)	24 (42.1)	0.008*
Married	15 (26.8)	32 (55.2)	28 (49.1)	
Divorced/widowed	13 (23.2)	4 (6.9)	5 (8.8)	
Education level				
Illiterate	5 (8.9)	4 (6.9)	2 (3.5)	0.000*
Elementary	17 (30.4)	9 (15.5)	3 (5.3)	
Middle	21 (37.5)	19 (32.8)	10 (17.5)	
Diploma	10 (17.9)	7 (12.1)	17 (29.8)	
University	3 (5.3)	19 (32.9)	25 (43.9)	
Occupation				
Housewife	10 (17.9)	4 (6.9)	3 (5.4)	0.000*
Employed	1 (1.8)	14 (24.1)	16 (28.1)	
Unemployed	42 (75)	14 (24.1)	13 (22.8)	
Other	3 (5.3)	26 (44.9)	25 (43.9)	
Family history of smoking	34 (60.7)	35 (60.3)	24 (42.1)	0.074*
Age of smoking initiation (years old)	4.74 ± 22.50	4.45 ± 20.24	–	0.010***
	(15–35)	(12–33)	–	
The amount of cigarettes used daily				
1 package	37 (66.1)	36 (62.1)	–	0.225*
2 packages	19 (33.9)	19 (32.8)	–	
3 packages	0	3 (5.1)	–	
Duration of smoking (years)	5.80 ± 12.48	9.52 ± 13.65	–	0.431***
	(2–30)	(2–45)	–	
Duration of schizophrenia (years)	6.61 ± 13.05	–	–	–
	(2–28)	–	–	
Type of anti-psychotic drug				
Typical	5 (8.9)	–	–	–
Atypical	46 (82.1)	–	–	
Both	5 (8.9)	–	–	
Scale of PANSS				
Positive symptoms	(12–35) 21.11 ± 5.60	–	–	–
Negative symptoms	(20–37) 27.05 ± 3.80	–	–	
General symptoms	(20–58) 38.57 ± 9.60	–	–	
Total score	(63–120) 86.77 ± 13.31	–	–	

*Chi-squared test/**ANOVA/***independent t-test, – not applicable.

Significant values are given in bold.

Concentrations of As, Cd, and Pb in blood serum samples and Tl in urine samples in the three investigated subgroups are presented in Table 2. The concentrations of Pb, As, Cd in blood serum and Tl in urine were the highest in the subgroup of cigarette smokers not diagnosed with schizophrenia, then in the cigarette smokers diagnosed with schizophrenia, and the lowest in the control group. However, concentrations were significantly higher (p < 0.05) only regarding mean Pb concentrations in blood serum samples in the cigarette smokers not diagnosed with schizophrenia group (mean value 5.16 μg/dL), comparing to the other two subgroups: mean value 3.83 μg/dL in the cigarette smokers diagnosed with schizophrenia group and mean value 3.43 μg/dL in the control group. No statistically significant differences were observed between cigarette smokers diagnosed with schizophrenia and control groups on all investigated heavy metals. In addition, the mean Cd and As concentrations in blood serum and Tl concentrations in urine were significantly higher (p < 0.05) in the cigarette smokers not diagnosed with schizophrenia than in the control group. No statistically significant differences (p > 0.05) between the pairs of cigarette smokers diagnosed with schizophrenia and control group, and between cigarette smokers not diagnosed with schizophrenia and cigarette smokers diagnosed with schizophrenia were observed.

**Table 2:** Concentrations of investigated heavy metals [μg/dL] in the studied groups; data are reported as mean ± standard error of the mean (SEM) (minimum–maximum).

Concentration [μg/dL]	Cigarette smokers diagnosed with schizophrenia	Cigarette smokers not diagnosed with schizophrenia	Control group (not diagnosed with schizophrenia and non-smokers)	p-value*	Pairs of groups**
Blood Pb concentration	3.83 ± 0.37 (0.4–9.5)	5.16 ± 0.35 (0.4–10.9)	3.43 ± 0.24 (0.0–7.4)	0.001	1–2: **p = 0.034** 1–3: p = 0.753 2–3: **p < 0.001**
Blood Cd concentration	0.74 ± 0.06 (0.0–2.53)	0.88 ± 0.07 (0–3.2)	0.63 ± 0.05 (0.0–1.8)	0.018	1–2: p = 0.246 1–3: p = 0.433 2–3: **p = 0.013**
Blood As concentration	8.04 ± 0.70 (23.7–0.5)	9.86 ± 1.01 (28.9–0.2)	5.98 ± 0.61 (0.0–19.4)	0.003	1–2: p = 0.369 1–3: p = 0.083 2–3: **p = 0.005**
Urinary Tl concentration	1.97 ± 0.23 (0.0–7.2)	2.55 ± 0.27 (0.2–11.2)	1.59 ± 0.19 (0.0–7.6)	0.014	1–2: p = 0.181 1–3: p = 0.487 2–3: **p = 0.010**

*μg/dL* micrograms per deciliter, *Pb* lead, *Cd* cadmium, *As* arsenic, *Tl* thallium.

*ANOVA.

**Groups are numbered as follows: 1—cigarette smokers diagnosed with schizophrenia, 2—cigarette smokers not diagnosed with schizophrenia, 3—Control (not diagnosed with schizophrenia and non-smokers).

Significant values are given in bold.

The results of the Pearson’s correlation analysis between clinical symptoms based on the PANSS criteria scale and Pb, Cd, and As concentrations in blood serum and Tl concentrations in urine are presented in Table 3. The Pearson’s correlation coefficients indicated a lack of the significant relationship (p > 0.05) between clinical symptoms based on the PANSS criteria and the heavy metals concentrations in the group of cigarette smokers diagnosed with schizophrenia group.

**Table 3:** The relationship between clinical symptoms of PANSS occurrence and the heavy metal concentrations [μg/dL] in smokers diagnosed with schizophrenia; data are reported as Pearson correlation coefficient (p-value).

Concentration [μg/dL]	Criteria scale positive and negative syndrome scale (PANSS)			
	Positive symptoms	Negative symptoms	General symptoms	Total score
Blood Pb concentration	0.0120 (0.378)	0.092 (0.502)	0.200 (0.138)	0.222 (0.100)
Blood Cd concentration	0.115 (0.397)	0.051 (0.706)	0.003 (0.980)	0.038 (0.782)
Blood As concentration	− 0.035 (0.798)	− 0.057 (0.677)	0.013 (0.925)	− 0.019 (0.888)
Urinary Tl concentration	− 0.024 (0.859)	− 0.081 (0.555)	0.123 (0.367)	0.056 (0.682)

*μg/dL* micrograms per deciliter, *Pb* lead, *Cd* cadmium, *As* arsenic, *Tl* thallium.

The results of the correlation between the duration of smoking and the investigated heavy metal concentrations in blood serum and urine are presented in Table 4. Pearson’s correlation coefficients revealed a statistically significant positive correlation (p < 0.001) in the cigarette smokers diagnosed with schizophrenia between blood Pb, blood As, and urine TI concentrations and the duration of cigarette smoking. In addition, in the cigarette smokers not diagnosed with schizophrenia, a statistically significant positive correlation (p < 0.01) was stated between the concentrations of all investigated heavy metals determined in blood and urine with the duration of smoking.

**Table 4:** The relationship between the duration of smoking and the heavy metal concentration [μg/dL] in groups of smokers and smokers diagnosed with schizophrenia; data are reported as Pearson correlation coefficient (p-value).

Concentration [μg/dL]	Duration of smoking	
	Cigarette smokers diagnosed with schizophrenia	Cigarette smokers not diagnosed with schizophrenia
Blood Pb concentration	0.702 (0.000)	0.568 (0.000)
Blood Cd concentration	0.168 (0.216)	0.372 (0.004)
Blood As concentration	0.860 (0.000)	0.846 (0.000)
Urinary Tl concentration	0.629 (0.000)	0.541 (0.000)

*μg/dL* micrograms per deciliter, *Pb* lead, *Cd* cadmium, *As* arsenic, *Tl* thallium.

The analysis of the occurrence of the symptoms related with heavy metal poisoning in the investigated subgroups is presented in Table 5. The frequency of appearance of the fatigue, paresthesia, ataxia, vertigo, memory deficits, seizures, depression, tinnitus, delusion and/or hallucination, emotional liability, mental retardation, and mood disorders symptoms in the group of cigarette smokers diagnosed with the schizophrenia subgroup were statistically significantly more frequent (p < 0.05) than in the other two investigated subgroups. Also, the frequency of symptoms of weakness, blurred vision, aggressiveness, abdominal pain, scalp hair loss, and sweating in the subgroups of cigarette smokers not diagnosed with schizophrenia and cigarette smokers diagnosed with schizophrenia was statistically significantly higher (p < 0.05) than in the control group (not diagnosed with schizophrenia and non-smokers). The frequency of constipation in the cigarette smoking subgroup not diagnosed with schizophrenia was statistically significantly higher (p < 0.05) than in the other two subgroups. The frequency of tremor symptoms in the cigarette smoking not diagnosed with schizophrenia subgroup was statistically significantly higher (p < 0.05) than in the control subgroup, and the frequency of insomnia symptoms in the cigarette smokers diagnosed with the schizophrenia subgroup was significantly higher (p < 0.05) than in the control group.

**Table 5:** Clinical symptoms occurrence of heavy metal poisoning in investigated subgroups; data reported as frequency (percentage).

Clinical signs of poisoning	Cigarette smokers diagnosed with schizophrenia	Cigarette smokers not diagnosed with schizophrenia	Control group (not diagnosed with schizophrenia and non-smokers)	p-value*
Weakness	34 (60.7)	29 (50)	9 (15.8)	**0.000**
Fatigue	46 (82.1)	11 (19)	4 (7)	**0.000**
Paresthesia	8 (14.3)	2 (3.4)	1 (1.8)	**0.013**
Ataxia	14 (25)	3 (5.2)	0	**0.000**
Vertigo	39 (69.6)	10 (17.2)	5 (8.8)	**0.000**
Blurred vision	4 (7.1)	6 (10.3)	0	**0.030**
Memory deficits	38 (67.9)	8 (13.8)	4 (7)	**0.000**
Tremor	9 (16.1)	3 (5.2)	0	**0.002**
Aggressiveness	18 (32.1)	23 (39.7)	7 (12.3)	**0.003**
Insomnia	12 (21.4)	19 (32.8)	6 (10.5)	**0.015**
Seizures	3 (5.4)	0	0	**0.034**
Depression	50 (89.3)	23 (39.7)	16 (28.1)	**0.000**
Tinnitus	4 (7.1)	0	0	**0.011**
Delusion and/or hallucination	51 (91.1)	0	0	**0.000**
Headache	3 (5.4)	5 (8.6)	3 (5.3)	0.705
Emotional liability	29 (51.8)	0	0	**0.000**
Mental retardation	3 (5.4)	0	0	**0.034**
Mood disorders	54 (96.4)	2 (3.4)	0	**0.000**
Wrist drop	1 (1.8)	0	0	0.327
Foot drop	0	0	0	–
Hearing loss	4 (7.1)	3 (5.2)	1 (1.8)	0.389
Muscle pain	4 (7.1)	9 (15.5)	6 (10.5)	0.358
Antisocial behaviors	2 (3.6)	0	0	0.106
Constipation	1 (1.8)	8 (13.8)	1 (1.8)	**0.007**
Nausea	0	0	0	–
Vomiting	0	0	0	–
Abdominal pain	3 (5.4)	6 (10.3)	0	**0.039**
Diarrhea	0	0	0	–
Loss of appetite	5 (8.9)	4 (6.9)	1 (1.8)	0.245
Renal failure	6 (10.7)	5 (8.6)	1 (1.8)	0.148
Anemia	9 (16.1)	12 (20.7)	5 (8.8)	0.200
Weight loss	1 (1.8)	3 (5.2)	2 (3.5)	0.617
High blood pressure	7 (12.7)	8 (13.8)	3 (5.3)	0.272
Scalp hair loss	24 (42.9)	27 (46.6)	11 (19.3)	**0.004**
Body hair loss	10 (17.9)	7 (12.1)	5 (8.8)	0.345
Sweating	12 (21.4)	11 (19)	1 (1.8)	**0.004**
Rashes	1 (1.8)	0	0	0.327
Dry skin	8 (14.3)	9 (15.5)	3 (5.3)	0.177
Mees’ lines	0	0	0	–
Palmar erythema	0	0	0	–
Acne	2 (3.6)	1 (1.7)	0	0.324
Pale face	1 (1.8)	0	0	0.327
Lead line	0	0	0	–

– Not applicable.

*Chi-square test/Fisher’s exact test.

Significant values are given in bold.

Mees' lines are white lines or bands that appear on the fingernails or toenails^37^.

The relation between clinical symptoms and the concentrations of investigated heavy metals is presented in Table 6 for Pb in blood, in Table 7 for Cd in blood, in Table 8 for As in blood, and in Table 9 for Tl in urine. In the subgroup of cigarette smokers, a significant correlation (p < 0.05) was revealed between schizophrenia, paresthesia, and ataxia symptoms and blood Pb concentrations, memory deficits and blood Cd concentrations, hearing loss and urine Tl concentrations, acne and blood Pb and Cd concentrations, blurred vision, scalp hair loss, and body hair loss and blood Pb and As concentrations, tinnitus and blood As and urine Tl concentrations, and tremor and dry skin and blood Pb and As and urine Tl concentrations. Also, in the subgroup of cigarette smokers, weakness, fatigue, paresthesia, vertigo, blurred vision, memory deficits, constipation, weight loss, and dry skin revealed a statistically significant correlation (p < 0.05) with blood As concentrations, renal failure with blood Pb and As concentration, ataxia with blood As and urine Tl concentrations, and scalp hair loss with blood Pb and As and urine Tl concentrations. In addition, in the control group, muscle pain had a statistically significant correlation (p < 0.05) with blood Pb concentrations, weakness with blood Cd concentrations, and scalp hair loss with blood As concentrations.

**Table 6:** Blood Pb concentrations [μg/dL] and the related heavy metal poisoning clinical symptoms in the studied subgroups; data reported as mean ± standard error.

Symptom	Cigarette smokers diagnosed with schizophrenia			Cigarette smokers not diagnosed with schizophrenia			Control group (not diagnosed with schizophrenia and non-smokers)		
	Yes	No	p-value*	Yes	No	p-value*	Yes	No	p-value*
	μg/dL								
Weakness	3.76 ± 0.48	3.94 ± 0.61	0.824	5.4 ± 0.508	4.8 ± 0.504	0.371	4.1 ± 0.490	3.3 ± 0.271	0.239
Fatigue	3.82 ± 0.41	3.87 ± 0.99	0.959	6.0 ± 1.001	4.9 ± 0.376	0.246	4.2 ± 0.537	3.3 ± 0.267	0.352
Paresthesia	5.97 ± 1.05	3.48 ± 0.38	**0.019**	5.8 ± 3.500	5.1 ± 0.353	0.735	–	–	–
Ataxia	5.37 ± 0.73	3.32 ± 0.41	**0.016**	7.2 ± 2.472	5.0 ± 0.354	0.177	–	–	–
Vertigo	4.11 ± 0.46	3.20 ± 0.62	0.267	6.1 ± 0.977	4.9 ± 0.374	0.193	4.7 ± 0.679	3.3 ± 0.250	0.083
Blurred vision	7.82 ± 0.67	3.52 ± 0.37	**0.002**	6.6 ± 1.263	4.9 ± 0.369	0.159	–	–	–
Memory deficits	3.85 ± 0.46	3.80 ± 0.66	0.958	6.6 ± 0.944	4.9 ± 0.372	0.095	4.1 ± 0.547	3.3 ± 0.268	0.413
Tremor	7.36 ± 0.40	3.16 ± 0.36	**0.000**	5.0 ± 2.170	5.1 ± 0.366	0.918			
Aggressiveness	4.29 ± 0.67	3.62 ± 0.45	0.408	5.6 ± 0.462	4.8 ± 0.505	0.288	2.7 ± 0.764	3.5 ± 0.253	0.290
Insomnia	4.30 ± 0.92	3.70 ± 0.41	0.521	5.3 ± 0.614	5.0 ± 0.447	0.724	3.7 ± 0.745	3.4 ± 0.260	0.658
Seizures	4.63 ± 1.94	3.79 ± 0.38	0.616	–	–	–	–	–	–
Depression	3.81 ± 0.40	4.05 ± 1.17	0.844	4.9 ± 0.568	5.2 ± 0.467	0.685	3.1 ± 0.390	3.5 ± 0.306	0.398
Tinnitus	6.04 ± 1.49	3.66 ± 0.38	0.103	–	–	–	–	–	–
Delusion and/or hallucination	3.75 ± 0.39	4.68 ± 1.23	0.485	–	–	–	–	–	–
Headache	2.98 ± 2.13	3.88 ± 0.38	0.594	5.0 ± 1.456	5.1 ± 0.377	0.933	3.3 ± 0.355	3.4 ± 0.254	0.851
Emotional liability	4.25 ± 0.49	3.38 ± 0.57	0.249	–	–	–	–	–	–
Mental retardation	2.57 ± 1.67	3.90 ± 0.38	0.426	–	–	–	–	–	–
Mood disorders	3.75 ± 0.38	5.95 ± 0.85	0.189	4.0 ± 1.700	5.2 ± 0.360	0.542	–	–	–
Hearing loss	2.15 ± 1.25	3.96 ± 0.39	0.216	4.8 ± 1.273	5.1 ± 0.377	0.833	–	–	–
Muscle pain	4.70 ± 1.30	3.77 ± 0.39	0.526	0.6 ± 0.065	0.6 ± 0.065	**0.004**	–	–	–
Antisocial behaviors	3.90 ± 1.90	3.83 ± 0.38	0.973	–	–	–	6.1 ± 0.851	5.0 ± 0.380	0.285
Constipation	–	–	–	–	–	–	–	–	–
Abdominal pain	4.53 ± 1.30	3.79 ± 0.39	0.661	5.3 ± 1.402	5.1 ± 0.364	0.880	–	–	–
Loss of appetite	3.90 ± 1.16	3.83 ± 0.40	0.956	3.2 ± 1.185	5.3 ± 0.360	0.144	–	–	–
Renal failure	5.87 ± 1.04	3.59 ± 0.39	0.060	7.4 ± 0.654	4.9 ± 0.374	**0.047**	–	–	–
Anemia	3.85 ± 0.91	3.83 ± 0.41	0.980	5.7 ± 0.952	5.0 ± 0.371	0.418	3.7 ± 0.764	3.4 ± 0.260	0.703
Weight loss	–	–	–	3.0 ± 1.500	5.2 ± 0.367	0.157	4.4 ± 0.855	3.4 ± 0.250	0.431
High blood pressure	4.14 ± 1.00	3.80 ± 0.41	0.767	5.8 ± 1.091	5.0 ± 0.375	0.465	4.9 ± 1.196	3.3 ± 0.245	0.142
Scalp hair loss	2.86 ± 0.54	4.56 ± 0.48	**0.023**	4.1 ± 0.398	6.0 ± 0.531	**0.007**	2.7 ± 0.506	3.5 ± 0.279	0.181
Body hair loss	2.21 ± 0.57	4.18 ± 0.42	**0.012**	4.4 ± 0.478	5.2 ± 0.405	0.234	2.9 ± 0.738	3.4 ± 0.288	0.570
Sweating	4.50 ± 0.92	3.65 ± 0.40	0.358	4.9 ± 0.915	5.2 ± 0.380	0.784	–	–	–
Dry skin	1.84 ± 0.60	4.16 ± 0.41	**0.006**	5.8 ± 1.071	5.0 ± 0.374	0.434	3.5 ± 1.327	3.4 ± 0.253	0.895
Acne	1.70 ± 0.30	3.91 ± 0.38	**0.003**	–	–	–	–	–	–

*μg/dL* micrograms per deciliter, *Pb* lead, *Cd* cadmium, *As* arsenic, *Tl* thallium.

– Not applicable.

*Independent t-test.

Significant values are given in bold.

**Table 7:** Blood Cd concentrations [μg/dL] and the related heavy metal poisoning clinical symptoms in the studied subgroups; data reported as mean ± standard error.

Symptom	Cigarette smokers diagnosed with schizophrenia			Cigarette smokers not diagnosed with schizophrenia			Control group (not diagnosed with schizophrenia and non-smokers)		
	Yes	No	p-value*	Yes	No	p-value*	Yes	No	p-value*
	μg/dL								
Weakness	0.81 ± 0.09	0.63 ± 0.07	0.160	0.9 ± 0.092	0.8 ± 0.125	0.639	0.8 ± 0.157	0. ± 08.0558	**0.035**
Fatigue	0.74 ± 0.07	0.72 ± 0.07	0.896	1.1 ± 0.235	0.8 ± 0.072	0.091	0.9 ± 0.191	0.6 ± 0.051	0.121
Paresthesia	0.64 ± 0.11	0.76 ± 0.07	0.498	0.7 ± 0.200	0.8 ± 0.089	0.648	–	–	–
Ataxia	0.68 ± 0.10	0.76 ± 0.07	0.574	1.4 ± 0.754	0.8 ± 0.075	0.516	–	–	–
Vertigo	0.75 ± 0.08	0.72 ± 0.08	0.809	1.0 ± 0.23	0.8 ± 0.086	0.479	0.8 ± 0.181	0.6 ± 0.051	0.267
Blurred vision	0.95 ± 0.25	0.72 ± 0.06	0.347	1.1 ± 0.382	0.8 ± 0.076	0.291	–	–	–
Memory deficits	0.65 ± 0.06	0.92 ± 0.13	**0.045**	1.1 ± 0.290	0.8 ± 0.075	0.249	0.9 ± 0.176	0.6 ± 0.050	0.065
Tremor	0.76 ± 0.36	0.73 ± 0.12	0.853	0.9 ± 0.230	0.8 ± 0.088	0.961	–	–	–
Aggressiveness	0.65 ± 0.09	0.78 ± 0.08	0.346	0.8 ± 0.074	0.9 ± 0.111	0.637	0.5 ± 0.137	0.6 ± 0.054	0.663
Insomnia	0.64 ± 0.06	0.77 ± 0.07	0.413	0.8 ± 0.071	0.9 ± 0.102	0.526	0.8 ± 0.190	0.6 ± 0.051	0.255
Seizures	0.71 ± 0.01	0.74 ± 0.06	0.897	–	–	–	–	–	–
Depression	0.72 ± 0.06	0.90 ± 0.17	0.372	0.8 ± 0.073	0.9 ± 0.122	0.515	0.6 ± 0.109	0.6 ± 0.060	0.453
Tinnitus	0.77 ± 0.11	0.74 ± 0.06	0.877	–	–	–	–	–	–
Delusion and/or hallucination	0.75 ± 0.06	0.58 ± 0.06	0.418	–	–	–	–	–	–
Headache	0.67 ± 0.09	0.74 ± 0.06	0.778	0.8 ± 0.178	0.8 ± 0.088	0.987	0.7 ± 0.268	0.6 ± 0.052	0.493
Emotional liability	0.72 ± 0.08	0.76 ± 0.09	0.787	–	–	–	–	–	–
Mental retardation	0.53 ± 0.32	0.75 ± 0.06	0.427	–	–	–	–	–	–
Mood disorders	0.75 ± 0.06	0.50 ± 0.30	0.455	1.2 ± 0.300	0.8 ± 0.087	0.432	–	–	–
Hearing loss	0.55 ± 0.24	0.75 ± 0.06	0.394	0.8 ± 0.033	0.8 ± 0.089	0.877	–	–	–
Muscle pain	0.47 ± 0.23	0.76 ± 0.06	0.233	1.1 ± 0.242	0.8 ± 0.084	0.171	0.6 ± 0.127	0.6 ± 0.052	0.791
Antisocial behaviors	0.35 ± 0.35	0.75 ± 0.06	0.223	–	–	–	–	–	–
Constipation	–	–	–	0.8 ± 0.094	0.8 ± 0.089	0.812	–	–	–
Abdominal pain	0.57 ± 0.14	0.75 ± 0.06	0.505	1.0 ± 1.392	0.8 ± 0.077	0.544	–	–	–
Loss of appetite	0.66 ± 0.22	0.75 ± 0.06	0.685	0.6 ± 0.345	0.9 ± 0.080	0.402	–	–	–
Renal failure	0.80 ± 0.20	0.73 ± 0.06	0.739	0.9 ± 0.158	0.8 ± 0.087	0.699	–	–	–
Anemia	0.85 ± 0.23	0.72 ± 0.06	0.448	0.9 ± 0.218	0.8 ± 0.086	0.530	0.8 ± 0.263	0.6 ± 0.051	0.222
Weight loss	–	–	–	0.7 ± 0.036	0.8 ± 0.089	0.696	0.7 ± 0.040	0.6 ± 0.052	0.776
High blood pressure	0.66 ± 0.17	0.75 ± 0.07	0.604	0.9 ± 0.306	0.8 ± 0.077	0.671	0.9 ± 0.287	0.6 ± 0.051	0.113
Scalp hair loss	0.77 ± 0.12	0.72 ± 0.06	0.717	0.8 ± 0.090	0.9 ± 0.125	0.319	0.6 ± 0.127	0.6 ± 0.052	0.681
Body hair loss	0.70 ± 0.23	0.75 ± 0.06	0.779	0.8 ± 0.135	0.8 ± 0.089	0.880	0.7 ± 0.174	0.6 ± 0.052	**0.475**
Sweating	0.68 ± 0.10	0.75 ± 0.07	0.636	0.8 ± 0.227	0.8 ± 0.089	0.939	–	–	–
Dry skin	0.74 ± 0.14	0.74 ± 0.07	0.992	0.9 ± 0.272	0.8 ± 0.077	0.820	0.6 ± 0.373	0.6 ± 0.053	0.982
Acne	0.10 ± 0.10	0.76 ± 0.06	0.043	–	–	–	–	–	–

*μg/dL* micrograms per deciliter, *Pb* lead, *Cd* cadmium, *As* arsenic, *Tl* thallium.

– Not applicable.

*Independent t-test.

Significant values are given in bold.

**Table 8:** Blood As concentrations [μg/dL] and the related heavy metal poisoning clinical symptoms in the studied subgroups; data reported as mean ± standard error.

Symptom	Cigarette smokers diagnosed with schizophrenia			Cigarette smokers not diagnosed with schizophrenia			Control group (not diagnosed with schizophrenia and non-smokers)		
	Yes	No	p-value*	Yes	No	p-value*	Yes	No	p-value*
	μg/dL								
Weakness	8.45 ± 0.91	7.42 ± 1.09	0.477	12.4 ± 1.485	7.26 ± 1.23	**0.009**	6.2 ± 0.989	5.9 ± 0.712	0.831
Fatigue	8.45 ± 0.91	7.42 ± 1.09	0.770	14.0 ± 2.443	8.88 ± 1.08	**0.046**	6.6 ± 1.847	5.9 ± 0.653	0.760
Paresthesia	10.91 ± 2.39	7.56 ± 0.70	0.093	23.1 ± 2.255	9.38 ± 0.99	**0.012**	–	–	–
Ataxia	8.76 ± 1.65	7.80 ± 0.76	0.560	18.6 ± 1.547	9.38 ± 1.03	**0.007**	–	–	–
Vertigo	8.23 ± 0.90	7.61 ± 1.04	0.687	14.2 ± 3.047	8.94 ± 1.02	**0.046**	6.9 ± 1.468	5.8 ± 0.668	0.619
Blurred vision	14.82 ± 3.34	7.52 ± 0.66	**0.006**	21.4 ± 1.977	8.52 ± 0.95	**0.000**	–	–	–
Memory Deficits	8.06 ± 0.95	8.0 ± 0.88	0.966	19.3 ± 2.014	8.34 ± 0.98	**0.000**	7.9 ± 1.537	5.8 ± 0.653	0.379
Tremor	13.28 ± 1.72	7.04 ± 0.68	**0.001**	16.8 ± 6.417	9.48 ± 1.00	0.107	–	–	–
Aggressiveness	8.87 ± 1.39	7.65 ± 0.79	0.419	10.9 ± 1.394	9.15 ± 1.41	0.392	3.5 ± 0.885	6.3 ± 0.682	0.141
Insomnia	10.12 ± 0.00	7.48 ± 0.69	0.122	12.0 ± 1.897	8.78 ± 1.17	0.130	4.5 ± 1.203	6.1 ± 0.675	0.423
Seizures	6.20 ± 2.31	8.15 ± 0.73	0.534	–	–	–	–	–	–
Depression	8.02 ± 0.75	8.20 ± 1.86	0.939	11.0 ± 1.592	9.10 ± 1.31	0.358	5.8 ± 0.900	6.0 ± 0.785	0.859
Tinnitus	13.52 ± 3.40	7.62 ± 0.68	**0.028**	–	–	–	–	–	–
Delusion and/or hallucination	7.80 ± 0.73	10.46 ± 2.10	0.282	–	–	–	–	–	–
Headache	4.43 ± 2.70	8.25 ± 0.71	0.221	16.7 ± 5.254	9.21 ± 0.97	0.227	4.8 ± 0.450	6.0 ± 0.654	0.655
Emotional liability	8.10 ± 1.02	7.98 ± 0.96	0.929	–	–	–	–	–	–
Mental retardation	2.83 ± 1.99	8.34 ± 0.71	0.075	–	–	–	–	–	–
Mood disorders	8.18 ± 0.71	4.40 ± 3.50	0.319	17.2 ± 3.655	9.60 ± 1.03	0.171	–	–	–
Hearing loss	3.12 ± 1.33	8.42 ± 0.72	**0.050**	14.5 ± 5.150	9.61 ± 1.03	0.290	–	–	–
Muscle pain	4.92 ± 1.69	8.28 ± 0.73	0.218	12.6 ± 2.601	35.10 ± 9.1	0.249	9.2 ± 1.903	± 60.635.0	0.070
Antisocial behaviors	4.65 ± 2.25	8.17 ± 0.71	0.354	–	–	–	–	–	–
Constipation	–	–	–	18.4 ± 1.761	8.49 ± 1.02	**0.000**	–	–	–
Abdominal pain	11.07 ± 2.12	7.87 ± 0.72	0.307	13.2 ± 3.245	9.47 ± 1.06	0.260	–	–	–
Loss of appetite	4.74 ± 1.29	8.37 ± 0.74	0.140	11.2 ± 3.992	9.76 ± 1.06	0.718	–	–	–
Renal failure	10.83 ± 3.17	7.71 ± 0.68	0.168	17.7 ± 2.454	9.12 ± 1.03	**0.016**	–	–	–
Anemia	6.69 ± 1.53	8.30 ± 0.78	0.401	11.5 ± 2.624	9.42 ± 1.09	0.402	6.0 ± 1.866	5.9 ± 0.657	0.969
Weight loss	–	–	–	1.93 ± 1.21	10.2 ± 1.049	**0.002**	5.5 ± 0.755	5.9 ± 0.649	0.895
High blood pressure	9.2 ± 44.03	80.0 ± 7.76	0.445	13. ± 48.342	9.28 ± 1.10	0.155	± 50.948.1	15.8 ± 0.644	0.340
Scalp hair loss	5.83 ± 0.92	9.70 ± 0.91	**0.005**	6.85 ± 1.07	12.7 ± 1.504	**0.001**	2.4 ± 0.775	6.8 ± 0.692	**0.004**
Body hair loss	4.63 ± 0.77	8.78 ± 0.79	**0.021**	8.98 ± 2.69	9.98 ± 1.10	0.752	2.8 ± 1.138	6.2 ± 0.658	0.119
Sweating	7.42 ± 1.72	8.21 ± 0.76	0.648	13.3 ± 2.846	9.04 ± 1.04	0.095	–	–	–
Dry skin	3.95 ± 0.78	8.72 ± 0.76	**0.015**	16.8 ± 2.285	8.58 ± 1.03	**0.002**	4.5 ± 2.030	6.0 ± 0.646	0.575
Acne	6.70 ± 4.30	8.09 ± 0.71	0.715	–	–	–	–	–	–

*μg/dL* micrograms per deciliter, *Pb* lead, *Cd* cadmium, *As* arsenic, *Tl* thallium.

– Not applicable.

*Independent t-test.

Significant values are given in bold.

**Table 9:** Urine Tl concentrations [μg/dL] and the related heavy metal poisoning clinical symptoms in the studied subgroups; data reported as mean ± standard error.

Symptom	Cigarette smokers diagnosed with schizophrenia			Cigarette smokers not diagnosed with schizophrenia			Control group (not diagnosed with schizophrenia and non-smokers)		
	Yes	No	p-value*	Yes	No	p-value*	Yes	No	p-value*
	μg/dL								
Weakness	1.90 ± 0.28	2.07 ± 0.40	0.729	2.5 ± 0.357	2.5 ± 0.413	0.944	1.9 ± 0.374	1.5 ± 0.213	0.428
Fatigue	1.96 ± 0.25	2.03 ± 0.66	0.905	3.8 ± 0.972	2.2 ± 0.236	0.147	1.4 ± 0.399	1.6 ± 0.200	0.888
Paresthesia	2.17 ± 0.57	1.93 ± 0.25	0.721	3.2 ± 1.055	2.5 ± 0.273	0.628	–	–	–
Ataxia	2.43 ± 0.51	1.81 ± 0.25	0.252	5.2 ± 1.700	2.4 ± 0.261	**0.020**	–	–	–
Vertigo	98.27 ± 1.0	1.95 ± 0.45	0.957	29.72 ± 3.0	2.4 ± 0.280	0.214	05.63 ± 2.0	1.5 ± 0.205	0.448
Blurred vision	2.85 ± 0.76	1.90 ± 0.24	0.296	3.2 ± 0.518	2.4 ± 0.297	0.361	–	–	–
Memory deficits	1.95 ± 0.30	2.01 ± 0.33	0.902	2.8 ± 0.510	2.5 ± 0.301	0.717	1.7 ± 0.350	1.5 ± 0.208	0.876
Tremor	3.78 ± 0.68	1.62 ± 0.21	**0.000**	3.0 ± 0.650	2.5 ± 0.283	0.702	–	–	–
Aggressiveness	2.25 ± 0.44	1.84 ± 0.27	0.409	2.6 ± 0.380	2.5 ± 0.372	0.880	1.8 ± 0.294	1.5 ± 0.216	0.633
Insomnia	2.49 ± 0.69	1.83 ± 0.23	0.243	2.6 ± 0.398	2.4 ± 0.359	0.737	2.0 ± 0.344	1.5 ± 0.204	0.412
Seizures	1.77 ± 1.25	1.98 ± 0.24	0.842	–	–	–	–	–	–
Depression	2.02 ± 0.25	1.54 ± 0.37	0.521	2.2 ± 0.355	2.7 ± 0.385	0.361	1.6 ± 0.268	1.5 ± 0.246	0.780
Tinnitus	4.37 ± 1.29	1.78 ± 0.21	**0.003**	–	–	–	–	–	–
Delusion and/or hallucination	1.86 ± 0.23	3.08 ± 1.11	0.135	–	–	–	–	–	–
Headache	1.83 ± 1.18	1.98 ± 0.24	0.890	2.7 ± 0.418	2.5 ± 0.293	0.798	1.8 ± 0.727	1.5 ± 0.197	0.712
Emotional liability	1.82 ± 0.31	2.13 ± 0.34	0.501	–	–	–	–	–	–
Mental retardation	0.53 ± 0.12	2.05 ± 0.24	0.141	–	–	–	–	–	–
Mood disorders	1.96 ± 0.24	2.63 ± 0.60	0.787	2.2 ± 0.000	2.5 ± 0.286	0.806	–	–	–
Hearing loss	0.54 ± 0.10	2.08 ± 0.24	**0.000**	2.1 ± 0.380	2.5 ± 0.288	0.697	–	–	–
Muscle pain	1.0 ± 0.32	2.04 ± 0.24	0.249	2.5 ± 0.389	2.5 ± 0.315	0.956	2.1 ± 0.549	1.5 ± 0.202	0.275
Antisocial behaviors	1.11 ± 0.01	2.00 ± 0.24	0.480	–	–	–	–	–	–
Constipation	–	–	–	3.5 ± 0.791	2.4 ± 0.280	0.155	–	–	–
Abdominal pain	3.43 ± 1.89	1.89 ± 0.22	0.500	2.1 ± 0.752	2.6 ± 0.290	0.585	–	–	–
Loss of appetite	1.10 ± 0.29	2.05 ± 0.25	0.243	1.2 ± 0.562	2.6 ± 0.285	0.181	–	–	–
Renal failure	2.97 ± 1.07	1.85 ± 0.22	0.346	1.9 ± 0.398	2.6 ± 0.291	0.517	–	–	–
Anemia	2.29 ± 0.59	1.91 ± 0.25	0.552	2.6 ± 0.446	2.5 ± 0.322	0.844	1.6 ± 0.551	1.5 ± 0.209	0.971
Weight loss	–	–	–	1.4 ± 0.177	2.6 ± 0.281	0.349	2.8 ± 0.027	1.5 ± 0.194	0.194
High blood pressure	1.0 ± 87.95	1.99 ± 0.24	0.868	± 35.843.0	2.4 ± 0.282	0.239	± 43.042.1	0.19 ± 1.54	0.298
Scalp hair loss	1.75 ± 0.33	2.13 ± 0.32	0.424	1.9 ± 0.238	3.0 ± 0.445	**0.040**	1.6 ± 0.309	0.2 ± 1.572	0.788
Body hair loss	1.59 ± 0.43	2.05 ± 0.26	0.449	1.7 ± 0.206	2.6 ± 0.306	0.276	1.5 ± 0.325	1.590 ± 0.2	0.948
Sweating	1.81 ± 0.44	2.01 ± 0.27	0.719	2.5 ± 0.370	2.5 ± 0.326	0.924	–	–	–
Dry skin	0.76 ± 0.11	2.17 ± 0.26	**0.000**	3.2 ± 0.498	2.4 ± 0.302	0.252	1.8 ± 0.316	0.2 ± 1.580	0.740
Acne	2.15 ± 1.05	1.96 ± 0.24	0.883	–	–	–	–	–	–

*μg/dL* micrograms per deciliter, *Pb* lead, *Cd* cadmium, *As* arsenic, *Tl* thallium.

– not applicable.

*Independent t-test.

Significant values are given in bold.

## Discussion

Our studies investigated the contents of As, Cd, Pb, and Tl in blood and urine samples among volunteers of patients diagnosed with schizophrenia who were continuous cigarette smokers and these metal concentrations were compared with analogous concentrations among cigarette smokers who were not diagnosed with schizophrenia, as well as with the control group who were not diagnosed with schizophrenia and non-smoking individuals. Best to our knowledge, studies on heavy metal concentrations among patients with schizophrenia were not very popular among researchers before. It might be related with the fact that such studies might raise ethical issues and they seem to be more difficult to perform in order of obtaining the results during questionnaire surveys.

However, the results of these studies might be useful in providing new knowledge on the correlations between inhalational exposure to cigarette smoke and public health. Especially in the aspect of early signs of diseases, among which disorders of the neurological system might be the first to appear^38^. Studies of Li et al.^39^ revealed that cigarette smoking might be associated with male cognitive impairment related to higher levels of iron (Fe), zinc (Zn), Pb, and Al in the human cerebrospinal fluid of active smokers.

It is also supported by the result of the other studies that revealed that cigarette smoking is very popular among patients with schizophrenia^23^. Among the reasons why individuals with schizophrenia smoke so much depend on psychosocial factors, issues related to the initiation of smoking in inpatient wards, and social affiliation, pleasure, dependence, boredom, and in order to accelerate/minimize the metabolism/side effects of antipsychotics are mentioned. Furthermore, the effects of nicotine on the schizophrenia symptoms must be considered as stated by Lucatch et al. due to the impact of nicotine in improving some of the disturbances in the dopaminergic, glutamatergic, or pathways affecting the gamma-aminobutyric acid (GABA) neurotransmitter^40^.

Ding and Hu^41^ stated that the three hypotheses that attempt to explain the high smoking rates among patients with schizophrenia are: self-medication, shared genetic propensity to smoking and the development of schizophrenia, and smoking as the trigger in the schizophrenia development. Dickerson et al.^42^ in their studies revealed that the prevalence of smoking was extremely high among individuals diagnosed with schizophrenia and that the disparity with participants without diagnosed psychiatric disorders and with the general population is increasing, thus suggesting additional investigations to address this public health issue.

The results obtained in our studies of high smoking rates among patients with schizophrenia were supported by various research^43–45^, which conclude that cigarette smoking is higher among individuals with schizophrenia than in the general population.

Regarding the PANSS scores, our studies were supported by the results of the investigations of Ma et al. who revealed a significant positive correlation between Mn concentrations in blood and the PANSS scores (total and negative scores) and a significant negative correlation between Ca blood concentrations and the PANSS scores (total and general scores)^46^. Also, the concentrations of magnesium (Mg), sodium (Na), selenium (Se), and Zn were negatively correlated with the total PANSS score. And finally, the authors investigated the intercorrelation of 11 elements between the case and the control group and found stronger and more positive correlations in the control group than in the case group^46^. Results of the studies of Misiak et al.^47^ indicated that cigarette smoking was associated with less severe negative and depressive symptoms, as well as delayed age of onset psychosis. These results stay in line with our findings.

Considering heavy metal contents in the body of cigarette smokers, the results presented in our studies were supported by the results of Ma et al.^48^ who revealed in their studies a significant association between higher serum concentrations of Pb and Cr with an elevated risk of schizophrenia in the study in patients with diagnosed drug-nave schizophrenia and drug-free patients were considered. The content of Pb could be related to the fact that Pb is mainly responsible for neurological adverse effects, and schizophrenia is one of these disorders. The results of Yılmaz et al.^49^ revealed that Cu levels in patients diagnosed with schizophrenia were lower than those of the controls. There was no significant difference between the groups in terms of Zn levels. However, the Cu/Zn ratio was lower in patients diagnosed with schizophrenia compared to that in controls. The results of Al-Fartusie et al.^27^ revealed a significant increase in Se, Cu, Ni, Cr, and manganese (Mn) in the blood, while a significant decrease in Zn, Mg, Pb, cobalt (Co), and Al was observed among patients diagnosed with schizophrenia compared to the control group. The study of Rahman et al.^50^ on heavy metal contents in hair from patients diagnosed with schizophrenia revealed that cigarette smoking did not have a significant effect among patients diagnosed with schizophrenia on metal contents, except for Ca (p = 0.020)^50^. On the other hand, other studies demonstrated a reduction in the serum concentrations of Zn, Cs, and Se that could increase the risk of schizophrenia due to the effect of the chemistry metabolism^51^. In their studies Cao et al.^52^ concluded that non-smoking patients diagnosed with schizophrenia had reduced concentrations of Mn and molybdenum (Mo), and increased concentrations of Ni in the blood comparing to the control group.

Our research also has some limitations. First issue might be related to the relatively small number of volunteers that we have recruited among patients diagnosed with schizophrenia. Also, we could investigate more heavy metals. Next issue might be related with the fact that in our research we did not consider active, called also firsthand smoke (FSH), nor passive smoking, that consists of second hand smoke (SHS) and third hand smoke (THS). SHS is a mixture of side stream and mainstream smoke, while THS consists of pollutants that settle indoors after smoking in closed environments^53^. Another issue is that we did not use DSM-IV/DSM-IV-TR versions in this study. The next one is that considering that we included primary schizophrenia in this study, it is suggested that secondary schizophrenia caused by drug use, seizure, or any other physical disease should be investigated in future studies. Finally, in our studies, the differences between geographical variability and gender differentiation were untouched. However, our investigation revealed that smoking affects the health of cigarette users and as neurological diseases are among the most popular related to heavy metal exposure, it is justified to continue this research area in further studies.

## Conclusions

Our results revealed a statistically significant correlation between blood and urine metal concentrations among cigarette smokers, both diagnosed and not diagnosed with schizophrenia, compared to the control group. Our results broaden the understanding of neuropsychiatric disorders related to exposure to heavy metals. Also, the results obtained in the current study justify further investigation in the perspective of public health as cigarette smoking is still increasing worldwide.

## Data Availability

The datasets generated and/or analyzed during the current study are not publicly available because the intellectual property is owned by the funding body. They may be available from the corresponding author on reasonable request containing the approval from the associated funding body.
